# *ATP2C1* gene mutations in Hailey–Hailey disease and possible roles of SPCA1 isoforms in membrane trafficking

**DOI:** 10.1038/cddis.2016.147

**Published:** 2016-06-09

**Authors:** M Micaroni, G Giacchetti, R Plebani, G G Xiao, L Federici

**Affiliations:** 1 School of Pharmaceutical Science and Technology, Dalian University of Technology, Dalian 116024, China; 2 Aging Research Center (Ce.S.I.), University ‘G. D'Annunzio' of Chieti-Pescara, Chieti 66100, Italy; 3 Department of Neuroscience, Imaging and Clinical Sciences, University ‘G. D'Annunzio' of Chieti-Pescara, Chieti 66100, Italy; 4 Department of Medical Oral and Biotechnological Sciences, School of Medicine and Health Sciences, University ‘G. D'Annunzio' of Chieti-Pescara, Chieti 66100, Italy

## Abstract

*ATP2C1* gene codes for the secretory pathway Ca^2+^/Mn^2+^-ATPase pump type 1 (SPCA1) localizing at the golgi apparatus. Mutations on the human *ATP2C1* gene, causing decreased levels of the SPCA1 expression, have been identified as the cause of the Hailey–Hailey disease, a rare skin disorder. In the last few years, several mutations have been described, and here we summarize how they are distributed along the gene and how missense mutations affect protein expression. SPCA1 is expressed in four different isoforms through alternative splicing of the *ATP2C1* gene and none of these isoforms is differentially affected by any of these mutations. However, a better understanding of the tissue specific expression of the isoforms, their localization along the secretory pathway, their specific binding partners and the role of the C-terminal tail making isoforms different from each other, will be future goals of the research in this field.

## Facts

- Mutations occurring on the *ATP2C1* gene clearly have no hotspots, although some mutations are redundant and the majority of missense mutations are spotted on specific exons.
- The C-terminal tails of two out of four SPCA1 isoforms display a sequence motif recognized by PDZ domains, potentially used to interact with different pools of protein and involved in different signaling pathways.
- SPCA1 has important roles in regulating membrane trafficking, not only as a Ca^2+^ pump able to trigger the Ca^2+^ influx into the lumen of the golgi apparatus (and of consequence the cytosolic peri-golgi Ca^2+^ concentration/signaling), but it also has a direct role in organizing cargo maturation/delivery from the golgi apparatus, which is imbalanced in cancer and other diseases.

## Open Questions

- Why mutations on the *ATP2C1* gene cause a different etiology between human and mouse? Does the overlap of *ATP2C1* gene with *ASTE1* gene have a role in regulating the SPCA1 expression in a different manner between species?
- Identification of proteins interacting with the C-terminal tails of the SPCA1 isoforms, and their possible role in mediating the function and the sub-organellar redistribution of the different SPCA1 isoforms in different cell types.
- Although SPCA1 is ubiquitously expressed in all the tissues, why mutations occurring on the *ATP2C1* gene are mostly affecting the skin?

The study of intracellular membrane trafficking is important for the understanding of cellular structure and organelle function, and the coordinated cellular activities within complex organisms. The intracellular transport can be divided into different phases, which include the synthesis of lipids and proteins in the endoplasmic reticulum (ER), their folding and quality control, transport from ER-to-golgi apparatus and across the golgi apparatus, and delivery of cargoes to their final destinations. The golgi apparatus also participates in the post-translational modifications (mostly glycosylation) of many proteins and lipids during their transport, and it is the central station of the intracellular secretory pathway.^1, 2^

The physiology of the secretory pathway and the golgi apparatus is finely regulated and maintained by pumps and channels that maintain the luminal pH/ion levels, making each sub-compartment of the golgi unique (i.e., the *cis*-side of the golgi apparatus is different from the *trans*-side).^3^ The secretory pathway Ca^2+^-ATPase pump type 1 (SPCA1) regulates the golgi luminal Ca^2+^ homeostasis, it is distributed along the secretory pathway membranes^4^ and ubiquitously expressed in all tissues.^5^

**Figure 1:**
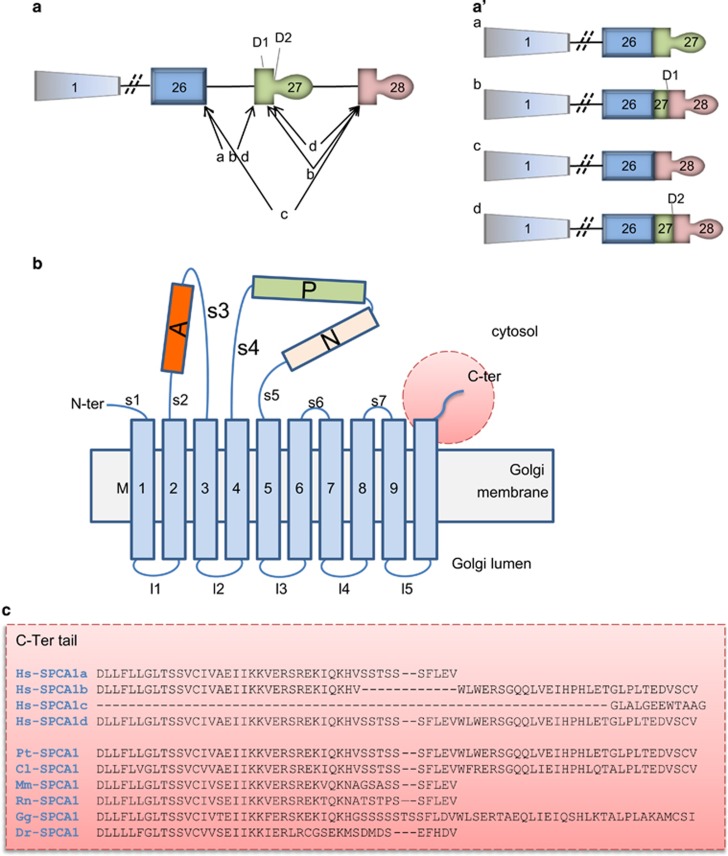
Representation of the *ATP2C1* gene alternatively spliced and the molecular structure of encoded SPCA1. (**a**) The *ATP2C1* gene consists of twenty-eight exons (represented by boxes), which are alternatively spliced as indicate by the internal 5′ donor splice sites, D1 and D2 generating four different mRNA. Diagonal lines illustrate the slicing patterns generating splice variants *ATP2C1*a-d. (**a′**) The *ATP2C1*a-d splice variants are schematically represented. (**a**) and (**a′**) are modified from Micaroni and Malquori.^50^ (**b**) Actuator domain (A), phosphorylation domain (P), nucleotide-binding domain (N) and 5 stalk helices (S) in the cytoplasm, and 10 transmembrane helices (M). This figure was adapted from Matsuda *et al.*^8^ (**c**) In gray is the exon 26, in yellow the exon 27, in green the exon 28. According to the present literature, the isoform SPCA1c seems not be coded in a protein. This isoform is missing the exon 27 coding for the transmembrane 10 (M10). Furthermore, this isoform is missing the possibility to have a cytosolic C-terminal tail where potential binding sites for other proteins is present, reinforcing the idea that this isoform is not functional. Hs-SPCA1a (NP_055197); Hs-SPCA1b (NP_001001487.1); Hs-SPCA1c (NP_001001485.2); Hs-SPCA1d (NP_001001486.1); Pt-SPCA1 (XP_001145788.1); Cl-SPCA1 (XP_534262.2); Mm-SPCA1 (NP_778190.3); Rn-SPCA1 (NP_571982.2); Gg-SPCA1 (XP_015137243.1); Dr-SPCA1 (XP_003200287). Hs: *Homo sapiens*; Pt: *Pan troglodytes*; Cl: *Canis lupus*; Mm: *Mus musculus*; Rt: *Rattus norvegicus*; Gg: *Gallus gallus*; Dr: *Danio rerio*

The human *ATP2C1* gene encoding for SPCA1 is located on chromosome 3q21 and consists of 28 exons.^6, 7^ Alternative processing at the 3′-end of the human *ATP2C1* pre-mRNA produces four distinct *ATP2C1* splice variants (corresponding to SPCA1a-d proteins; Figure 1a), namely (i) SPCA1a from the splicing of exon 26 to exon 27 with the translation stop codon located in exon 27 producing a protein of 919 amino acids; (ii) SPCA1b which contains 939 amino acids and results from splicing of exons 27 to 28 following activation of the internal 5′-splice donor site D1; (iii) splicing of exons 26–28 gives rise to SPCA1c, which has 888 amino acids; (iv) splicing at internal site D2 in exon 27 to exon 28 gives rise to SPCA1d, which is the largest variant with 949 amino acids (Figure 1a′).

The resulting four SPCA1 protein isoforms differ in their C-terminal cytosolic tails and are organized in actuator domain (A), phosphorylation domain (P), nucleotide-binding domain (N), 5 stalk helices (S) in the cytoplasm, and 10 transmembrane helices (M) (Figure 1b).^8^ The C-terminal tails of each SPCA1 isoforms, unique to each alternatively spliced product (Figure 1c), present characteristics for potential specific functions that we will discuss later. The alternative splicing is not present in other species where we have a single SPCA1 (Figure 1c).

**Figure 2:**
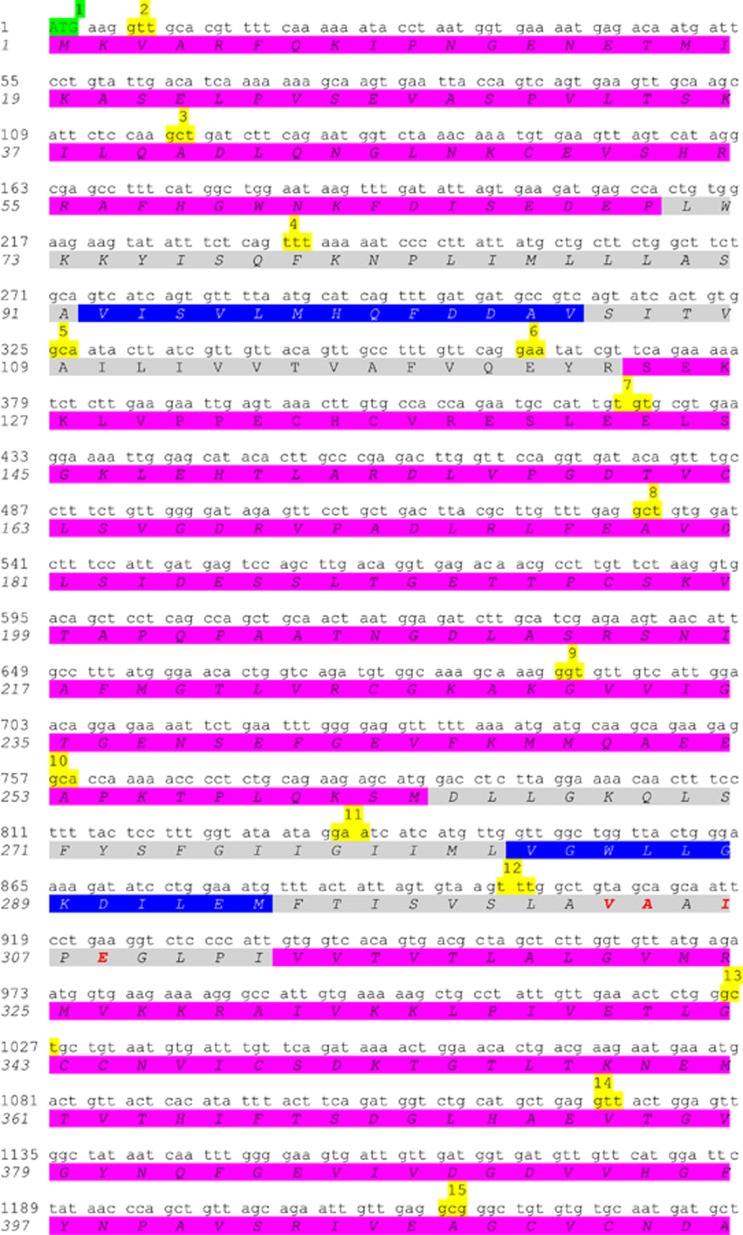

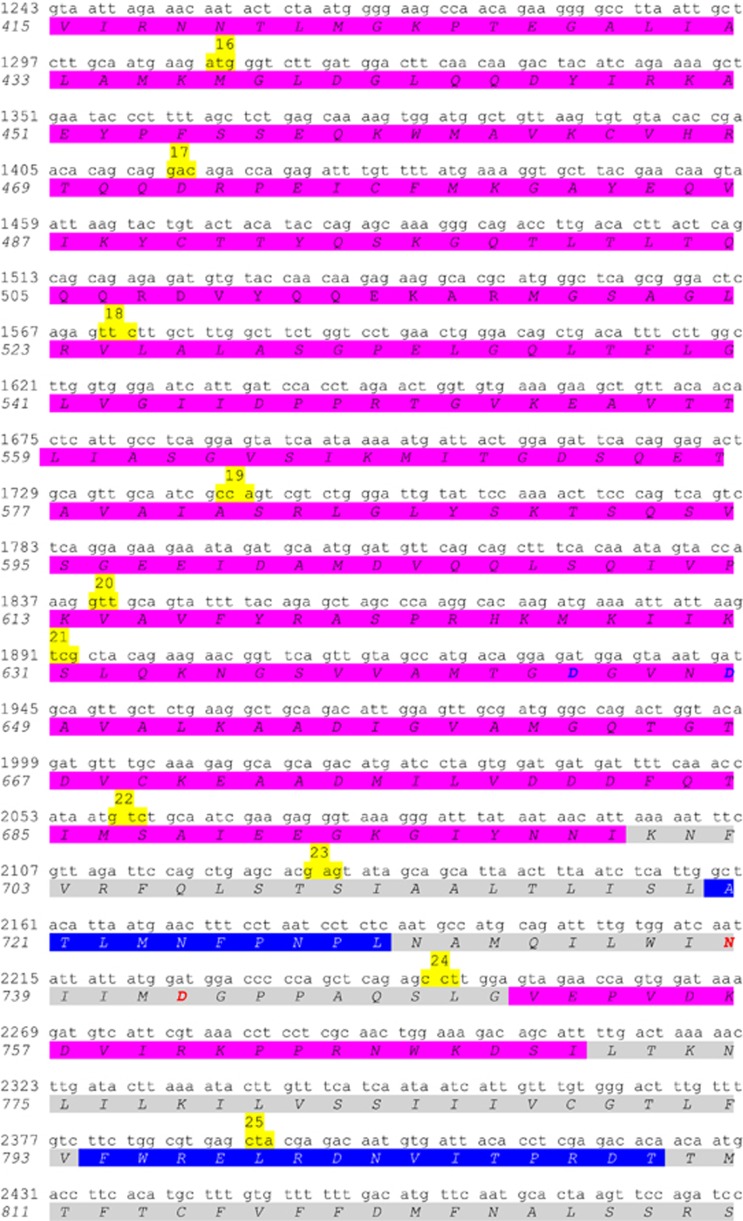

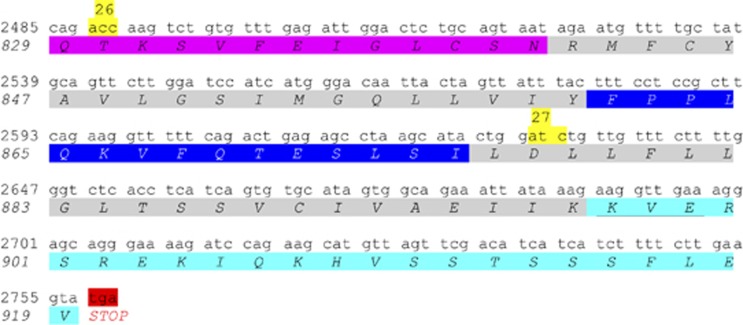
Identification of transmembrane domains *vs* cytosolic or luminal domains of SPCA1. For simplicity, SPCA1a (NP_055197) has been represented; other isoforms have identical sequence until the C-terminal tail. The highlighted codons in green and in red are the starting and stop codon, respectively. Highlighted in yellow is the first codon of each corresponding numbered exon. Highlighted in gray is the transmembrane domain. The highlighted amino acids in purple are cytoplasmic, whereas those highlighted in blue are luminal. The highlighted amino acids in azure represent the cytosolic C-terminal tail (underlined is the KXD/E motif putative COPI-binding site). The bold typed red and blue amino acids are the residues directly involved in the binding of Ca^2+^/Mn^2+^ and Mg^2+^, respectively. Of note, the putative COPI-binding motif KXD/E is just below the M10 domain. The prediction of these domains/residues was solved by similarity (UniProt database)

A schematic representation of the *ATP2C1* gene sequence and the relative encoded SPCA1 protein sequences is reported in Figure 2. Here, we show where the exons start/finish (codons highlighted in yellow), which is the corresponding cytosolic (purple), transmembrane (gray) and luminal (blue) portion of SPCA1, as well as the C-terminal tail (azure).

## Hailey–Hailey Disease

Mutations of the *ATP2C1* gene have been reported in Hailey–Hailey disease (HHD) patients,^6, 7^ originally described by the Hailey brothers (Hugh Edward and William Howard) in 1939.^9^ HHD is also known as familial benign chronic pemphigus^10^ or familial benign pemphigus.^11^ The prevalence of HHD is estimated to be 1:50 000.^12^ HHD is a monoallelic genetic disorder inherited in an autosomal dominant pattern,^6, 7^ meaning that one copy of the altered gene is sufficient to cause the disorder. Comparison between genotype and phenotype failed to show a clear correlation between the nature of the mutation and the clinical features of HHD (age of onset, severity, progression). Extensive inter-familial and intra-familial variation was noted in clinical features, as well as between families sharing the same mutation.^13^ Mutations in the *ATP2C1* gene reduce the amount of functional SPCA1. This abnormality impairs cells ability to store Ca^2+^ normally. For unknown reasons, this abnormal Ca^2+^ storage affects keratinocytes more than other cells types. The abnormal regulation of Ca^2+^ impairs many cell functions, including cell adhesion. As a result, keratinocytes do not stick tightly to one another, which causes the epidermis to become fragile and less resistant to minor trauma. Because the skin is easily damaged, it develops raw, blistered areas, particularly in skin folds where there is moisture and friction.

Recent observations shed new light on a possible role for altered Ca^2+^ homeostasis and responsiveness in HHD keratinocytes in creating an early defect in differentiation process due to a reduced production of involucrin.^14^ Keratin expression was also delayed in acantholytic epidermal segments of HHD skin.^15^ Because of decreased Ca^2+^ stored in the lumen of the golgi apparatus of HHD keratinocytes, involucrin mRNA degraded in response to abnormally raised cytosolic Ca^2+^.^16^ From dermatological perspectives, the HHD patients have been treated with topical steroid preparations to help outbreaks. Several drugs (antibiotics, antifungals, corticosteroids, etc.) keep under control the progression of the disease but are ineffective for severe chronic or relapsing forms. Some HHD patients found relief in laser resurfacing that burns off the top layer of the epidermis, allowing healthy non-affected skin to regrow in its place.

## Mutations on the Human *ATP2C1* Gene

Here, we review the literature about the mutations occurring on the *ATP2C1* gene, and give a comprehensive view of where these mutations fall along the gene sequence. Furthermore, we focus our attention on why some missense mutations affect SPCA1 efficiency without affecting the protein levels and address our interest to amino acidic residues that could probably have a role in the correct functioning of the protein and are not yet fully characterized. Finally, we focus our attention on the possible role of three different cytosolic tails of the SPCA1a, SPCA1b, and SPCA1d isoforms, and their potential for binding different partners.

Several mutations have been reported on the *ATP2C1* gene in HHD patients, some of which were redundant even if no evident hotspots have been recognized so far. In Table 1 we summarize all the mutations reported so far, mentioning their localization along the gene sequence, the kind of mutation as well as the resultant change in the amino acid sequence. All the mutations generating truncated protein, which supposedly do not have a complete functionality or precise sub-organellar localization or are destabilized and therefore arguably destined for degradation, are indicated as premature termination codon (PTC).

After careful reviewing of literature using PubMed and the Chinese Biological Medicine Database (http://www.sinomed.ac.cn/zh/), we listed a total of 166 unique mutations on the *ATP2C1* gene that have been reported in HHD to date. Of them, ~55% lead to PTC, supporting the possibility that haploinsufficiency of *ATP2C1* is a prevalent mechanism for the dominant inheritance of HHD. Twenty-four (~14%) were nonsense mutations, fifty-nine (~36%) were deletion/insertion mutations (of which five (~4%) are in-frame deletion and insertion mutations), thirty-four (~20%) were splice-site mutations, and forty-nine (~30%) were missense mutations (Table 1). Thus, many mutations predict the absence, or a marked reduction of the mutated *ATP2C1* product via nonsense-mediated mRNA decay. Non-conservative amino acid changes in functional domains of the molecule are highly conserved between golgi Ca^2+^ pumps from different species, and between other Ca^2+^-ATPases. In fact, some of these mutations occur at amino acid residues conserved between SPCA1 and SERCA1 (the ER ATPase Ca^2+^ pump type 1); the latter has previously been studied by site-directed mutagenesis and the relevant mutations were shown to abrogate SERCA1 function.^17^

Mutations are scattered along the *ATP2C1* gene without apparent clustering, showing a substantial allelic heterogeneity, and are distributed all over the encoded sequence (Supplementary Figure 1), as well as in the intron splice sites generating alternative splicing and/or truncated proteins. Mutations affect all domains of the resulting protein. Rarely, mutations seem to be addressed to the 3′ end of the gene (namely the last 3 exons/introns), where the alternative splicing generates the four different isoforms of SPCA1.

Few nonsense mutations were frequently reported in different families worldwide (reports with at least three cases): 115*C*>*T* causing R39X;^6, 18, 19^ 457*C*>*T* causing R153X;^7, 20, 21, 22, 23, 24, 25^ 1402*C*>*T* causing R468X;^7, 26, 27, 28^ 1516*C*>*T* causing Q506X;^29, 30^ and 2395*C*>*T* causing R799X.^18, 25, 31, 32, 33, 34^ The above single residues represent ‘hotspots' for mutations, and notably, they all occur at *CpG* sites where the possibility of mutations is higher.^35^ The fact that the majority of the mutations cause PTC through frameshift or single-base-pair substitution points to haploinsufficiency as the prevalent mechanism for the dominant inheritance of HHD.^6, 7^ However, the possibility that at least some mutations cause the disease through a dominant negative mechanism cannot be excluded.^13^

Interestingly, only one mutation was found in the most 3′ region (exons 27–28) of the gene where differential splicing generates the four different transcripts.^22^ The single reported mutation, located in exon 27, was a nonsense mutation (2660*C*>*A*), which results in a stop codon (S887X) on the last transmembrane domain (M10). The mutation was found in a patient with a family history of HHD and suffering from classical HHD symptoms.^22^ Notably, this mutation caused the loss of the C termini of the SPCA1a, SPCA1b and SPCA1d isoforms but not of SPCA1c that does not include exon 27. Thus, SPCA1c, the shortest of the isoforms, misses the M10, and therefore, its stability and consequent ability to functionas a Ca^2+^ pump is rather questionable.^36^ Nevertheless, if SPCA1c was effectively expressed, we can conclude that it is not functional enough to overcome the loss of the other three isoforms at least in the skin of the patient presenting the mentioned S887X mutation.^22^ Remarkably, this observation suggests that the mRNA encoding for SPCA1c is probably not translated as previously suggested.^37^

Furthermore, the mutation on exon 27 causes a reduction in SPCA1a, SPCA1b and SPCA1d of 33, 52 and 63 amino acids, respectively, resulting in a partial truncation of the M10 and of the full cytoplasmic tail, similarly to what happens in SPCA1c (888 amino acids). Of consequence, this mutation is predicted to determine an incomplete expression and degradation of these SPCA1 isoforms thus resembling SPCA1c. Hereafter, we will no more consider SPCA1c in this discussion.

Splice variants differ in their C-terminal tail sequence (Figure 1c) and this is likely to be important for the functionality of the pump. Indeed, the C-terminal tail could have a role in mediating interactions with cytoplasmic effectors for intracellular signaling or for targeting the single isoforms to specific sub-organellar localization. Presently, no cytoplasmic interacting proteins have been found for the SPCA1 C-terminal domain. However, specific interactions of different SPCA1 tails with proteins involved in membrane trafficking (i.e., golgi matrix, Ca^2+^-binding proteins, Arf/Rab family members, cytoskeleton, etc.) could support a specific role for C-terminal tail in the differential distribution of the protein at sub-organellar level. This would in turn make them unique not only in triggering the Ca^2+^ influx into the golgi apparatus, but also in mediating different cytoplasmic signaling, thus orchestrating membrane trafficking at different levels along the secretory pathway. Obviously, these speculations need to be experimentally proven.

No further mutations have been discovered in the 3′-end of the *ATP2C1* gene. In other words, once the *ATP2C1* gene is mutated (independently if missense, nonsense, insertion and/or duplication or part of the primary sequence) the functionality of all the isoforms is equally compromised in HHD patients, and at least in the skin, levels of SPCA1 appear to be not sufficient. However, up-regulation of SERCA2 (the ER ATPase Ca^2+^ pump type 2) has been recently shown to (at least partly) compensate for the decreased levels of SPCA1 in HHD.^38^

Therefore, although SPCA1 is ubiquitously expressed, is there differential expression of the isoforms? Is the compensatory role for non-functional or depleted SPCA1 by other pumps tissue specific? Is there a general mechanism? If so, how does it work? Even if some tentative explanations have been given,^38, 39^ these open questions need answers to fully understand the role and distribution of the SPCA1 isoforms.

## Missense Mutations on the *ATP2C1* Gene

**Figure 3:**
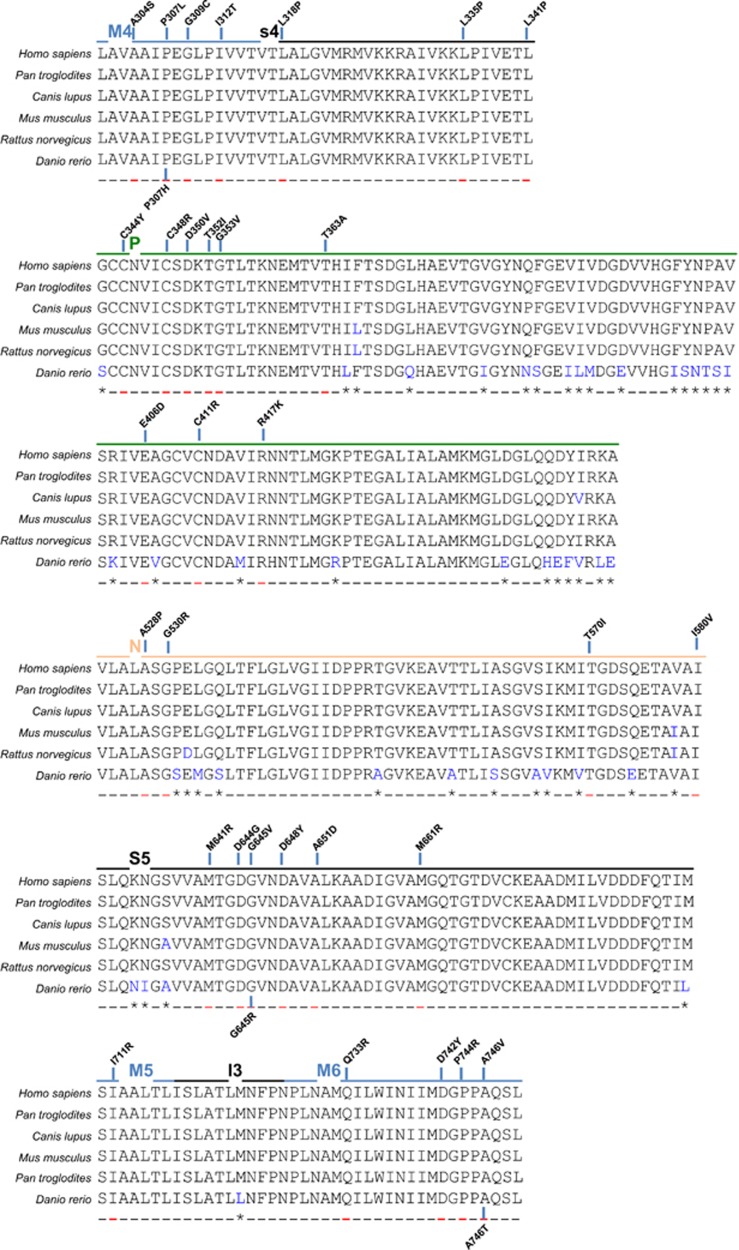
Missense mutations mostly affect critical SPCA1 domains. Most of the missense mutations localize on exons 12, 13–16, 18, 21, and 23 of *ATP2C1* gene coding for the indicated protein domains (M4, P, N, S5, M5-M6) crucial for the protein enzymatic activity and Ca^2+^/Mn^2+^ binding. In human HHD patients the missense mutations cause the indicated amino acid changes, supporting a crucial role for them in functionality of the SPCA1. Of note, the mutations localize on highly conserved residue/codon between the considered species, highlighting the importance of those encoded amino acids. In blue are indicated the different amino acids compared to the human sequence, in red where the mutations occur. Alignments were obtained from Ensembl database as described in Figure 1

Missense mutations generate single amino acid substitution, not leading to a PTC. A quick view on where the missense mutations occur along the *ATP2C1* gene sequence gives an idea of their preferential distribution in few exons. In particular, almost two-thirds (63%) of the missense mutations (31/49) localize in only five exons (exon 12, 13, 18, 21, and 23; Table 1). These exons encode for M4, P, ATP and M5/M6 domains, respectively (Figure 3). Amino acids located on M4, M5 and M6 (together with M8) transmembrane domains are critical for Ca^2+^/Mn^2+^ binding. It is not surprising that affecting residues in these regions, either directly involved in Ca^2+^-binding or compromising the stability or structure of the protein, may cause a severe effect on SPCA1 functionality, also without a reduction in its levels. Looking at the conservation across species we found high level of homology of these exons and conservativeness of the resulting amino acid coded by the codons where missense mutations occur (Figure 3), confirming their critical role.

Several residues have been reported to have a critical and direct role in Ca^2+^/Mn^2+^ binding, which are conserved throughout species^40^ and also in SPCA2, the homolog of SPCA1.^41^ Missense mutation D778A in the yeast homolog (PMR1) resulted in a loss of function mutant apparently defective for the transport of both Ca^2+^ and Mn^2+^, whereas mutant Q783A displayed a differential sensitivity consistent with the selective loss of Mn^2+^ transport.^42^ In a similar study, G783 and V335 in PMR1 showed conformation-sensitive packing at the cytoplasmic interface, suggesting that these residues are important in organizing the entry gate for Mn^2+^ ions.^43^ Missense mutation can also cause a change in protein conformation affecting possible interaction of SPCA1 with other proteins, like cofilin, which has been found to be crucial in recruiting actin to mediate the cargo sorting that interact directly with SPCA1 nucleotide domain through an interaction mediated by Ca^2+^ ions.^44^

Remarkably, these examples highlight how a possible missense mutation can affect SPCA1 resulting not only in a less efficient pump, but also accounting for its diminished ability to interact with other proteins. In some HHD patients this scenario results in a severe etiology, comparable to the situation where mutations on *ATP2C1* gene cause PTC, but in the presence of unaffected levels of total protein expression.

## *ATP2C1* Gene: Human *Versus* Murine

Another aspect to highlight is the different etiology occurring between humans and mice as a consequence of *ATP2C1* mutations. In humans, HHD rarely degenerates in skin cancer although squamous cell carcinoma and basal cell carcinoma arising in lesions of HHD have been described in the literature.^45, 46, 47^ However, which is the relationship, if any, between skin cancer development and HHD has not been deeply investigated. To our knowledge, there are no reports of melanoma or non-cutaneous malignant neoplasms associated with HHD.

On the other side, heterozygotic mice exhibit no evidence of skin lesions indicative of HHD, meanwhile generated skin cancer in adult.^48, 49^ Null mutant embryos exhibited growth retardation, failure of neural tube closure, but normal hematopoiesis and cardiovascular development, resulting in embryonic lethality.^48^ At subcellular level, golgi membranes of Spca1^−/−^ embryos were dilated, had fewer stacked leaflets, and were expanded in amount, consistent with increased golgi biogenesis.^48^ Increased golgi-associated vesicles and a marked reduction of ribosomes on rough ER were also observed.^48^ Coated pits, junctional complexes, desmosomes, and basement membranes appeared normal in mutant embryos, indicating that processing and trafficking of proteins in the secretory pathway was not massively impaired.^48^ However, apoptosis was increased, possibly the result of secretory pathway stress, and a large increase in cytoplasmic lipid was observed in mutant embryos, consistent with impaired handling of lipids by the golgi.^48^ Possibly, reduction of the proper levels of Spca1 resulted in alteration in the activity of the glycosyl-transferases due to a decreased accumulation of Mn^2+^ in the golgi lumen, which could contribute to the secretory pathway stress as well as apoptosis due to a decreased of Ca^2+^ handling.

Recently, an important difference between human and murine genomes that could explain the differential outcome of mutations in the two species has been reported.^50^ The *Atp2c1* gene in mice expresses only one isoform of the gene, not presenting alternative splicing events at the 3′-end of the gene like the human counterpart. The last exon of the *ATP2C1* human gene overlapped in the antisense direction with the last exon of the *ASTE1* (also named *HT001*) gene, which codes for a protein of unknown function.^50^ *ATP2C1*/*ASTE1* overlap (not present in the murine genome) could have a role in regulating isoform expression, or more largely, the full SPCA1 expression.^50^ This sort of regulation could have in turn a protective role that is imbalanced once one of the two alleles is mutated in humans, whereas not in mice.^50^

## Putative Interacting Domains in the C-terminal Cytosolic Tails of SPCA1 Isoforms

As suggested above, different SPCA1 isoforms due to their unique C termini could have a different pattern of distribution and functional roles. The primary sequence of the C-terminal tail of each isoform is reported in Figure 1c. An intriguing possibility is that the uniqueness of their function is a consequence of the interaction with different protein partners. It is well known that many resident proteins display in their tails, motifs like sorting signals responsible for retrieval or retention. For instance, the C-terminal KKXX motif, identified in many ER-localized type I membrane proteins, can function as an ER-retention signal to retrieve ER-resident membrane proteins from the golgi via a direct interaction with coat protein complex I (COPI) coatomer.^51, 52, 53^ Another example is the semi-conserved (F/L)-(L/V)-(S/T) motif, which has been shown to function as a golgi-retention signal by interacting with COPI vesicles via Vps74p and thereby maintaining the steady golgi localization of glycosyl-transferases in yeast.^54^ Recently, a conserved retention function and COPI-binding ability for the KXD/E motif has been shown in the golgi apparatus through the evolution in different species and in many endomembrane proteins.^55, 56^

**Figure 4:**
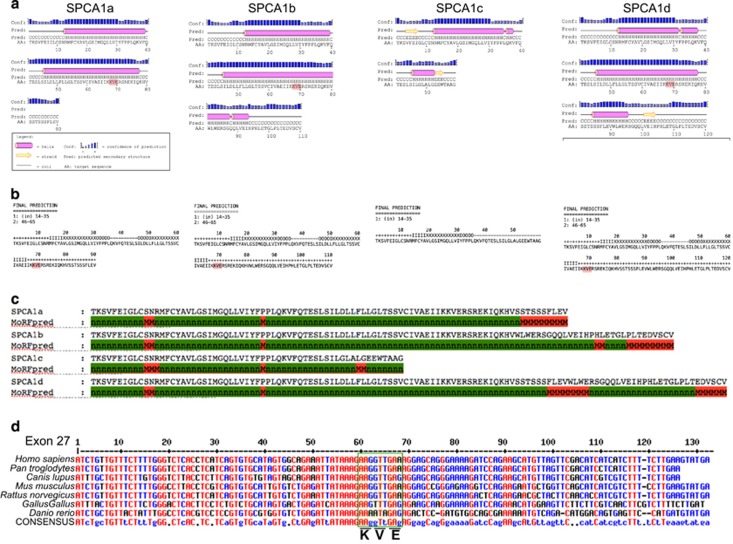
Bioinformatics analysis of SPCA1 isoforms C-terminal region. **(a**) Secondary structure propensities in the proteins' regions encoded by terminal exons were predicted with the program PSIPRED.^92^ Highlighted in pink is the corresponding sequences in the KXD/E motif. Predictions indicate that this motif is located in a helical extension spanning outside transmembrane helix M10 (see also **b**). Interestingly this helix is predicted to have different length in the three isoforms where it is present (**a**, **b** and **d**). (**b**) Transmembrane topology prediction was performed using the algorithms MEMSAT3 (ref. 93) and TMHMM2 (http://www.cbs.dtu.dk/services/TMHMM) yielding very similar results. In the isoforms SPCA1a, SPCA1b and SPCA1d, two transmembrane helices are predicted by both algorithms, the first (M9) with cytosolic-lumenal orientation and the second (M10) with opposite orientation. In the SPCA1c isoform a single cytosolic-lumenal transmembrane helix (M9) is predicted. Shown are results from MEMSAT3 where ‘+' stands for cytosolic residue, ‘−' for lumenal residue and I, X and O for transmembrane residues (I close to cytosolic, X central and O close to lumenal). (**c**) The MoRFPred algorithm^94^ was used to predict, in the different isoforms, regions of the protein that are prone to interact with protein partners, due to disorder-to-order transitions, here highlighted in red. The EDSCV motif found at the C-terminal of isoforms b and d matches the consensus sequence specifically recognized by class I PDZ domains. The other regions predicted in isoforms ‘a' and ‘d' may be recognized by unknown protein partners. Highlighted in red are the putative residues of binding motifs. (**d**) The exon 27 encoding for most of the M10 domain and the C-terminal tail where putative binding motifs are located. The KXD/E is highly conserved. Sequence of *Homo sapiens* was compared to the four species of mammals chosen (Ensembl database. ID: *Homo Sapiens* ATP2C1-011 ENST00000428331; *Pan troglodytes* ATP2C1-201 ENSPTRT00000028742; *Canis lupus* ATP2C1-201 ENSCAFT00000031759; *Mus musculus* ATP2C1-005 ENSMUST00000038118; *Rattus norvegicus* ATP2C1-201 ENSRNOT00000018175) and two non-mammalian (Ensembl database. ID: *Gallus gallus* ATP2C1-201 ENSGALT00000018903; *Danio rerio* ATP2C1-001 ENSDART00000084528)

Using bioinformatics tools (see Figure 4 legend), we analyzed the SPCA1 sequences in the areas corresponding to the terminal exons 26–28 in terms of secondary structure propensity (Figure 4a), predicted transmembrane helices topology (Figure 4b), and interaction with putative protein partners (Figure 4c). We found that the KXD/E motif is present in isoforms SPCA1a, SPCA1b, and SPCA1d, in the cytosolic tail immediately after the M10 (Figure 4c) in a region that is predicted to be a helical extension of M10 in all three isoforms (compare Figures 4a and b). This motif is also highly conserved throughout different species suggesting functional importance (Figure 4d).

Therefore, SPCA1 is potentially involved in the interaction with COPI. This reinforces the idea that SPCA1 could be involved in regulating local fusion/fission of cargo domain/vesicles carrying proteins along the secretory pathway, triggering the cytosolic Ca^2+^ level in the peri-golgi membranes, where Ca^2+^-binding proteins are temporarily recruited to organize and regulate the membrane fusion/fission, a fundamental process for cargo progression.^4, 57^

The analysis of the potential of C-terminal tail residues to interact with protein partners also highlighted additional sequences, which are differentially present in the four isoforms (Figure 4c). In particular, the isoforms SPCA1b and SPCA1d both end with the C-terminal sequence EDVSCV, which is predicted to be a protein interaction site (Figure 4c). By inspecting the literature we realized that this motif perfectly reproduces the structural features that are necessary for recognition by class I PDZ domains: (i) being at the C-terminal of a protein and (ii) matching the consensus for the last three residues S/T-*X*-Φ-COO−,^58^ where *X* is any residue and Φ is a hydrophobic residue (often a valine). Thus, this motif is strongly predicted to interact with the PDZ domains of other proteins.^59^ Several proteins containing the PDZ domain can potentially interact with the tail of SPCA1b and SPCA1d modulating the function of the pump. Interestingly, evidences that the C-terminal tail of SPCA1 could interact with the golgi matrix arise from a recent observation of the interaction of C-terminal valine-bearing cargoes moving through the golgi apparatus by binding to GRASP55 and GRASP65, two golgi matrix proteins that contain PDZ domains.^60^ PDZ domain recognition of the C-terminal tail is involved in golgi maintenance and in cargo progression.^60, 61^ The SPCA1 isoforms present a valine residue as last amino acid residue in the C-terminal position (Figures 1c and 4c).

Also the FLEV motif present at the C-terminal end of SPCA1a and in the cytoplasmic tail of SPCA1d is predicted to be a protein binding motif (Figure 4c). Interestingly, this motif is also present in rat calsequestrin (CASQ; AAA75480). CASQ regulates Ca^2+^-binding in the sarcoplasmic reticulum in cardiac muscle cells. However, this domain is not conserved between the human and murine proteins. This unique feature of the human protein could favor specific protein interactions by SPCA1 isoforms to mediate signaling and trigger the modulation of Ca^2+^ levels in the interested subcellular compartment. In the case of CASQ the interactors could be triadin and junctin.^62^ Analogs could have the same role in the golgi apparatus for SPCA1, working as Ca^2+^-mediated sensing probes to regulate the pump activity.

The above observations reinforce the hypothesis for a functional role of each different C-terminal tail in mediating interaction with different proteins.

## Importance of SPCA1 in Membrane Trafficking: What is the Possible Role of each Isoform?

In addition to the maintenance of intra-golgi Ca^2+^ and Mn^2+^, which has direct effects on Ca^2+^-dependent proteases^63^ and Mn^2+^-dependent glycosyltransferases^64^ (see also below), there is also evidence that SPCA1 may affect cytosolic Ca^2+^ signaling.^65, 66, 67, 68, 69^ Ca^2+^ released from the golgi leads to a localized increase in cytosolic Ca^2+^ that stimulates vesicle fusion, thus contributing to intra-golgi transport of cargo,^70^ consistent with the distribution of SPCA1 in the golgi apparatus.^4^ Thus, maintenance of luminal Ca^2+^ stores by SPCA1 may enable Ca^2+^ signaling events that directly affect the transport of cargo-containing domains from ER to the golgi apparatus and from there to their final destinations.^3^ If so, a deficiency in this signaling function likely contributes to the mislocalization of proteins in response to a decreased SPCA1 activity/level.^71, 72, 73^

Furthermore, effects on the ER were also observed in mammalian cells. Depletion of SPCA1 affected degradation of a mutant glycoproteins via ERAD, although it did not affect degradation of a non-glycoprotein substrates.^71^ Signaling pathways that mediate ER stress responses were intact and functional; however, SPCA1-deficient cells were highly sensitive to treatment with tunicamycin or thapsigargin, both of which cause ER stress and activate ER stress responses.^71^ Evidences of golgi stress, secondary effects on the ER, and stress responses were categorized as survival responses or apoptotic responses.^72^

Although the role of SPCA1 in the Ca^2+^ homeostasis of the golgi apparatus and its role in membrane trafficking has been well described and accepted,^70, 73^ the role of single isoforms still needs to be investigated. Possible differential distribution along the secretory pathway at sub-organelle level as well as at tissue level remains completely uninvestigated too. SPCA1 isoforms differ from each other in their C-terminal tails that is not directly involved in the regulation of Ca^2+^-binding. However, many Ca^2+^-binding proteins are recruited on the golgi membranes during the trafficking pulse (cPLA2, calmodulin, SNARE cofactors, etc.).^3^ These proteins are recruited on the golgi membranes through a local release of Ca^2+^ from the golgi apparatus and they should be released as soon as they fulfill their own function.^3^ The recruited Ca^2+^-binding proteins are addressed to the so-called lateral rims (or non compact zones) of the golgi stacks and in both the *cis*- and *trans*-golgi area where SPCA1 is clearly localized.^4^ Whether SPCA1 C terminus tails are involved in establishing temporary contacts with Ca^2+^-binding recruited proteins remains unknown.

SPCA1 is also able to transport Mn^2+^ ions into the golgi with high affinity, an ability that SERCAs do not possess. As Mn^2+^ is not used for signaling like Ca^2+^, the main reason for removing it from the cytosol is to prevent Mn^2+^ toxicity.^74^ Several proteins within the pathway require either Ca^2+^ and Mn^2+^, or other divalent ions to function as metal cofactors, such as amino-peptidase P,^75^ pro-protein convertases^76^ and sulfotransferarese.^77^ Even if there is no differential efficiency in the removal of ions by each SPCA1 isoform, potential differential sub-organellar localization of the isoforms may reflect the pump activity involved in different Ca^2+^/Mn^2+^-dependent pathways.

The inefficient removal of Ca^2+^/Mn^2+^ from the cytosol by SPCA1, with consequent decreased accumulation in the golgi, causes alteration in intracellular signaling and post-transcriptional protein modifications. Mn^2+^ is a fundamental cofactor of the golgi resident enzymes mannosidases.^78^ Reduced removal of Ca^2+^ from the cytosol during the arrival and the passage of cargo proteins to and through the golgi apparatus, significantly reduces physiological protein secretion.^4^

Furthermore, SPCA1 seems to have a direct role in organizing the cargo sorting at the *trans*-golgi network^79, 80^ and subsequent departure.^81^ Functional SPCA1 is important for insulin-like growth factor receptor (IGF1R) processing in basal-like breast cancer, and inhibition of SPCA1 ‘may offer an alternative strategy to direct inhibitors of IGF1R and attenuate the processing of other proprotein convertase substrates…'.^82^ However, which of the SPCA1 isoforms, if any, works at this level still needs to be defined.

## Membrane Trafficking in HHD Keratinocytes

In the skin of HHD patients, one of the altered clusters of proteins affected by non-functional or depleted SPCA1 are the adhesion proteins. The loss of one functional copy of the *ATP2C1* gene in HHD is characterized by the development of skin lesions and an associated loss of cell–cell adhesion.^6, 7^ Proteins necessary for desmosome formation (as well as extracellular matrix components) are likely to be directly involved in cellular interactions and are affected in SPCA1^+/−^ cells.^83^ Desmosomes are molecular complexes of cell adhesion proteins and linking proteins that attach the cell surface adhesion proteins to intracellular keratin cytoskeletal filaments. Desmoglein and desmocollin, two components of the desmosome, are members of the cadherin family of cell adhesion molecules. They are transmembrane proteins that bridge the space between adjacent epithelial cells by way of homophilic binding of their extracellular domains to other desmosomal cadherins on the adjacent cell.^83^ Both have five extracellular domains and have Ca^2+^-binding motifs. Defects in anchoring to cytoskeletal constituents can also reflect possible intracellular morphological defects as observed in golgi apparatus.^84^

Another important finding relating SPCA1 depletion and protein secretion in keratinocytes comes from a study on involucrin (a protein that makes up the cornified envelope of keratinocytes and is expressed in response to increased cytosolic Ca^2+^).^14^ Involucrin is expressed at lower levels in cells from HHD patients or cells treated with SPCA1 RNAi when compared to normal keratinocytes.^14^ Decreased level of involucrin was caused by lower involucrin mRNA levels in HHD keratinocytes; decreased involucrin mRNA, in turn, was caused by increased rates of involucrin mRNA degradation.^14^ Ca^2+^-sensitive involucrin AP-1 promoter activity was increased, both in HHD keratinocytes and in RNAi experimental model, suggesting compensatory promoter up-regulation in the face of increased mRNA degradation.^14^ Thus, transport/secretion of desmosomes components are impaired by SPCA1 depletion.

Further observations revealed that claudins are also regulated by SPCA1.^85^ Claudins are a family of proteins that are the most important components of the tight junctions, establishing a barrier that controls the flow of molecules in the intercellular space between the cells of an epithelium. In SPCA1-depleted keratinocytes protein levels of components of desmosomes, adherens and tight junctions did not show marked changes except for claudin1 and 4 that were increased.^85^

Whether and which SPCA1 isoforms are implicated in the regulation of specific cell adhesion protein trafficking is unknown. Nevertheless, the reason why desmosomes/tight junctions are affected more in the skin than in other epithelial tissues has to be investigated.

## Conclusions

Distribution of SPCA1 isoforms along the secretory pathway in different cell types could lead to a better understanding of which isoform is doing what, when, and where. Considering that SPCA1 is ubiquitously expressed, the fundamental role of this protein in regulating the Ca^2+^/Mn^2+^ physiology^86^ and membrane trafficking in different cell types/tissues could be due to specific clusters of SPCA1 isoforms. Further investigations to clarify the role of each SPCA1 isoforms will benefit the understanding of the biology of secretion in health and disease, and will impact on several pathological conditions.

## Figures and Tables

**Table 1:** Mutations on the *ATP2C1* gene reported in the literature

**Exon/Intron**	**Nucleotide change**	**Mutation**	**Number**^a^	**Codon**^b^	**Effect**	**Domain**^c^	**References**
Exon 2	28delG/ins24bp^d^	Deletion/insertion	1		PTC	N-ter	^6, 23^ d
Exon 2^d^	115C>T	Nonsense	4	R39X	PTC	N-ter/s1	^6, 18, 19^
Intron 2	117+2T>G	Donor splice	1		PTC(?)	N-ter/s1	^95^
Intron 2	118-2A>G	Acceptor splice	1		PTC	N-ter/s1	^96^
Intron 2	118-1G>A	Acceptor splice	3			N-ter/s1	^22^ e^,^^23, 97^ d^,^ f
Exon 3^d^	134delG	Deletion	1	45GfsX1	PTC	N-ter/s1	^98^
Exon 3	163C>T	Nonsense	3	R55X	PTC	N-ter/s1	^31, 97^ d^,^ f^,^^99^
Exon 3	168delC	Deletion	1	?	PTC	N-ter/s1	^31^
Exon 3	180G>A^d^	Nonsense	1	W60X	PTC	N-ter/s1	^97^
Exon 3	185delAGTT	Deletion	1	62KfsX34	PTC	N-ter/s1	^28^
Exon 3	212delT	Deletion	1	71LfsX26	PTC	M1	^100^^g^
Intron 3	235-2A>G	Acceptor splice	2			M1	^101, 102^
Exon 5	335delT	Deletion	1	111LfsX19	PTC	M2	^23^
Intron 5	360+1G>A	Donor splice	1			M2	^33^
Intron 5	360+1G>C	Donor splice	1		Skip exon 5	M2	^24^
Intron 5	360+2T>A	Donor splice	2		Skip exon 5	M2	^103^
Intron 5	361–1G>A^d^	Acceptor splice	1		PTC/skip exon 6	M2	^19^
Intron 5^d^	361-2A>G^d^	Acceptor splice	1		PTC/loss exon 6	M2	^104^
Exon 6	366T>A	Nonsense	1	Y122X	PTC	M2	^105^
Exon 7	457C>T	Nonsense	8	R153X	PTC	A	^7, 20, 21, 23, 24, 25^ e^,^^106^
Exon 7	490delT	Deletion	1	163LfsX24	PTC	A	^13^
Exon 7	519insA	Insertion	2	173LfsX3	PTC	A	^13, 33^
Exon 7	520delC	Deletion	1	174RfsX14	PTC	A	^24^
Intron 7	531+2T>A^d^	Donor splice	1			A	^18^
Exon 8	602C>T^h^	Missense	2	P201L^h^		A	^6^ d^,^^13^
Exon 8^d^	635C>A	Nonsense	1	S212X	PTC	A	^96^
Exon 8	661A>C^d^	Missense	1	T221P		A	^87^
Exon 8	681dupA	Insertion	1	227KfsX13	PTC	A	^24^
Intron 8	688-1G>A	Acceptor splice	1			A	^13^
Exon 9	689G>A	Missense	1	G230D		A	^107^
Exon 9	705delA	Deletion	1	235TfsX12	PTC	A	^108^
Exon 9	745C>T	Nonsense	1	Q249X	PTC	S3	^13^
Exon 10	767insCCCT	Insertion	1	256TfsX42	PTC	S3	^7^
Exon 10	775C>T	Nonsense	1	Q259X	PTC	S3	^105^
Exon 10	806T>G	Missense	1	L269R		M3	^105^
Exon 10	832G>A^i^	Missense/insertion	2	278GfsX22		M3	^91^
Intron 10	832+3A>T	Donor splice	2			M3	^13^
Intron 10	832+2T>C	Donor splice	1		Skip exon 10	M3	^20^
Intron 10^d^	833-1G>A^d^	Acceptor splice	1			M3	^6^
Exon 11	836insT	Insertion	1	279IfsX19	PTC	M3	^7^
Exon 11	854G>A	Nonsense	1	W285X	PTC	l2	^95^
Intron 11	899+1G>T	Donor splice	1		PTC	M4	^24^
Intron 11	899+1G>C	Donor splice	2		PTC	M4	^109^
Exon 12	910G>T	Missense	2	A304S		M4	^7^ j^,^^20^
Exon 12	920C>T	Missense	1	P307L		M4	^104^
Exon 12	920C>A	Missense	1	P307H		M4	^36^
Exon 12	923delAAG	Deletion	1	308delE^k^		M4	^28^
Exon 12	925G>T	Missense	1	G309C		M4	^13^
Exon 12	932del21bp^d^	Deletion	1	311del^d^		M4	^18^
Exon 12	935T>C	Missense	1	I312T		M4	^110^
Exon 12	950del9bp/ins24bp^l^	Deletion/insertion	1	318-320del/ins^l^		S4	^20^
Exon 12	953T>C	Missense	1	L318P		S4	^7^
Exon 12	956delC	Deletion	1	319AfsX3	PTC	S4	^24^
Exon 12	1001delA	Deletion	1	333KfsX12	PTC	S4	^13^
Exon 12	1004T>C	Missense	1	L335P		S4	^111^
Exon 12	1022T>C	Missense	1	L341P		S4	^13^
Intron 12	1024+1G>A^d^	Donor splice	1		PTC/skip exon 12	S4	^21^
Exon 13	1031G>A^d^^,^ h	Missense	1	C344Y^d^^,^ h		P	^6^
Exon 13	1042T>C	Missense	1	C348R		P	^105^
Exon 13	1045delT	Deletion	1	348CfsX6	PTC	P	^13^
Exon 13	1049A>T	Missense	3	D350V		P	^28^
Exon 13	1055C>T^d^	Missense	1	T352I		P	^112^
Exon 13	1058G>T^d^	Missense	1	G353V		P	^30^
Exon 13^d^	1067delC	Deletion	1	356TfsX3	PTC	P	^96^
Exon 13	1068del16bp^m^	Deletion	1	356TfsX60	PTC	P	^113^
Exon 13	1085insA	Insertion	1	363TfsX11	PTC	P	^29^
Exon 13	1087A>G	Missense	1	T363A		P	^114^
Exon 13	1089delTCAC	Deletion	4	363TfsX21	PTC	P	^13, 23, 28, 115^
Exon 14	1218G>C^d^	Missense	1	E406D	Skip exon 14	P	^19^
Exon 15	1231T>C	Missense	1	C411R		P	^13^
Exon 15^d^	1250G>A^d^	Missense	3	R417K		P	^32^
Intron 15	1308+1G>A	Donor splice	1			P	^36^
Intron 15	1309-1G>A	Acceptor splice	1			P	^13^
Intron 15	1309-4a>t/1309-2a>g	Acceptor splice	1		Skip exon 16	P	^7^
Exon 16	1327C>T^d^	Nonsense	1	Q443X^d^	PTC	P	^6^
Exon 16	1388T>G	Missense	1	V463G		P	^20^
Exon 16	1402C>T	Nonsense	4	R468X	PTC	P	^7, 26, 27, 28^
Exon 16	1413G>C	Missense	1	Q471H		?	^110^
Intron 16	1415-2A>C	Acceptor splice	1		PTC/skip exon 17	?	^104^
Exon 17	1413del28bp^n^	Deletion	2	472DfsX14	PTC	?	^116^
Exon 17	1431T>A	Nonsense	1	C477X	PTC	?	^117^
Exon 17	1455delA^d^	Deletion	1	485QfsX1	PTC	N?	^118^
Exon 17	1462del^d^^,^^o^	Deletion	1	488del^o^		N	^104^
Exon 17	1469G>T	Missense	1	C490F		N	^31^
Exon 17	1508delCTCA^d^	Deletion	1	503TfsX32	PTC	N	^18^
Exon 17	1510C>T	Nonsense	1	Q504X	PTC	N	^36^
Exon 17	1516C>T	Nonsense	4	Q506X	PTC	N	^29, 30^ d
Exon 17	1523delAT	Deletion	2	508DfsX23	PTC	N	^97, 104^
Exon 17	1566delCA	Deletion	1	522LfsX9	PTC	N	^7^
Intron 17	1570+2T>C	Donor splice	1		PTC	N	^24^
Exon 18	1582G>C	Missense	1	A528P		N	^23^
Exon 18	1588G>C	Missense	1	G530R		N	^95^
Exon 18	1685C>G	Nonsense	3	S562X	PTC	N	^6^ d^,^^13, 28^
Exon 18	1709C>T^d^^,^ h	Missense	2	T570I^d^^,^ h		N	^6^
Exon 18	1723delG	Deletion	1	574QfsX24	PTC	N	^13^
Exon 18	1738A>G	Missense	2	I580V		N	^13, 31^
Intron 18	1694-1G>A	Acceptor splice	1			N	^6^
Exon 19	1751T>C	Missense	1	L584P		N	^21^
Exon 19	1782delAGTC	Deletion	1	593SfsX5	PTC	N	^119^
Exon 19	1816C>T	Nonsense	1	Q606X	PTC	N	^13^
Intron 19	1839+2insT^d^	Donor splice	1		PTC	N	^23^
Intorn 19	1840-1G>C	Acceptor splice	1			N	^98^
Exon 20	1854G>A^d^	Missense	1	R619K		N	^87^
Exon 20	1869delG	Deletion	1	623RfsX2	PTC	N	^33^
Exon 20	1874delA	Deletion	1	M626X	PTC	N	^120^
Exon 20	1875delG	Deletion	1	M626X	PTC	s5	^7^
Intron 20	1890+1delGTGAG/ins	Donor splice	1			s5	^22^ e
Intron 20	1891-1G>T	Acceptor splice	1			s5	^121^
Exon 21	1897C>T	Nonsense	1	Q633X	PTC	s5	^122^
Exon 21	1914del/ins^d^	Deletion/insertion	1	638Vfs10X	PTC	s5	^123^ d
Exon 21	1922T>G	Missense	1	M641R		s5	^7^
Exon 21^d^	1931A>G	Missense	1	D644G		s5	^33^
Exon 21	1933G>A	Missense	1	G645R		s5	^7^
Exon 21	1934G>T^d^	Missense	1	G645V		s5	^124^
Exon 21	1942G>T	Missense	1	D648Y		s5	^104^
Exon 21	1952C>A	Missense	1	A651D		s5	^105^
Exon 21	1982T>G	Missense	1	M661R		s5	^113^
Exon 21	1983delG	Deletion	1	661MfsX14	PTC	s5	^7^
Exon 21	2023delA^d^	Deletion	1	675MfsX	PTC	s5	^18^
Exon 21	2025delG	Deletion	1	675MfsX2	PTC	s5	^105^
Intron 21^d^	2058(-17C>T)^d^	Acceptor splice	1		PTC	s5	^97^
Intron 21^d^	2058-1G>C^d^	Acceptor splice	1			s5	^28^
Intron 21	2058-1G>A	Acceptor splice	1		Skip exon 22	s5	^7^
Exon 22	2068G>T	Nonsense	1	E690X	PTC	s5	^110^
Exon 22	2111insA	Insertion	1	704RfsX23	PTC	M5	^20^
Exon 22	2126C>T	Missense	3	T709M		M5	^7, 28, 107^
Intron 22	2127+1G>A^d^	Donor splice	2		Skip exon 23 (?)	M5	^28^ d^,^^125^ d
Intron 22^d^	2126(+5G>A)^d^	Donor splice	1		PTC	M5	^97^
Exon 23	2132T>G	Missense	1	I711R		M5	^31^
Exon 23	2141T>A	Nonsense	1	L714X	PTC	M5	^23^
Exon 23	2164insACAT	Insertion	1	722LfsX6	PTC	l3	^122^
Exon 23	2198A>G	Missense	1	Q733R		M6	^31^
Exon 23	2215delATT	Deletion	1	739delI		M6	^20^
Exon 23	2224G>T	Missense	1	D742Y		M6	^13^
Exon 23	2227delG^d^	Deletion	1	743GfsX8	PTC	M6	^6^
Exon 23	2231C>G	Missense	2	P744R		M6	^7^ p^,^^20^
Exon 23	2235insC	Insertion	1	746AfsX10	PTC	M6	^107^
Exon 23	2236G>A^d^	Missense	1	A746T^d^		M6	^18^
Exon 23	2237C>T^d^	Missense	1	A746V		M6	^34^
Intron 23	2243+2T>C	Donor splice	1		PTC	M6	^104^
Exon 24	2246T>G	Missense	1	L749R		M6	^20^
Exon 24	2251delGT	Deletion	1	751VfsX5	PTC	s6	^126^
Exon 24	2264delA	Deletion	2	755DfsX17	PTC	s6	^25, 33^
Exon 24	2303delAC	Deletion	2	768DfsX4	PTC	s6	^7, 20^
Exon 24	2339delTTGT^d^	Deletion	1	780LfsX3	PTC	M7	^19^
Exon 24	2357delTT	Deletion	1	786IfsX10	PTC	M7	^7^
Exon 24	2365G>A	Missense	1	G789R		M7	^13^
Exon 24	2371delTTGT	Deletion	4	791LfsX12	PTC	M7	^7, 20, 127^ d
Exon 24	2374delTTTG	Deletion	10	792FfsX10	PTC	M7	^6^ d^,^^7, 13, 23, 31, 117, 128, 129^ d^,^^130^
Exon 24	2375delTTGT	Deletion	2	792FfsX4	PTC	M7	^102, 104^
Exon 24	2384G>A	Nonsense	1	W795X	PTC	l4	^22^ e
Exon 25	2395C>T	Nonsense	14	R799X	PTC	l4	^16^ d^,^^25, 31, 32, 33, 34^ d
Exon 25^d^	2412delT	Deletion	1	803IfsX7	PTC	l4	^102^
Exon 25	2416C>T	Nonsense	1	R806X	PTC	l4	^13^
Exon 25	2422delAC	Deletion	2	808TfsX10	PTC	l4	^33^
Exon 25	2445del10bp	Deletion	3	814CfsX7	PTC	M8	^131^
Exon 25	2454delT	Deletion	1	818FfsX6	PTC	M8	^31^
Exon 25	2454dupT	Insertion	1	D819X	PTC	M8	^28^
Exon 25	2460delG	Deletion	1	820MfsX4	PTC	M8	^21^
Exon 25	2468A>C^d^	Missense	2	A823E		M8	^97^
Exon 26	2529delGT	Deletion	1	843MfsX27	PTC	M9	^7^
Exon 26	2558del10bp^q^	Deletion	1	853MfsX17	PTC	M9	^31^
Exon 26	2593C>T^d^	Nonsense	2	Q865X^d^	PTC	l5	^6, 112^
Exon 26^d^	2597A>C	Missense	1	K866T		l5	^96^
Intron 26	2630-1delG	Acceptor splice	2			M10	^6^ d^,^^13^
Exon 27	2660C>A	Nonsense	1	S887X	PTC	M10	^22^^e^

Appendix 1							
Exon 23	2130T>C^d^	Polymorphism	1	S710S^d^		M5	^87, 88^

Appendix 2				
**Number**	**Mutation**	**%**	**PTC**	**%**
59	Deletion/insertion	35.54	55	59.78
24	Nonsense	14.46	24	26.09
49	Missense	29.52	0	0.00
34	Acceptor/donor splice	20.48	13	14.13
166			92	55.42

Appendix 3													
Normal													
p.309	G	G	P	I	V	V	T	V	T	L	A	L	p.320
c.925	GGT	CTC	CCC	*ATT*	*GTG*	*GTC*	*ACA*	*GTG*	*ACG*	*CTA*	*GCT*	*CTT*	c.960
c.925	*GGT*	*CTC*	*CTA*	*GCT*	*CTT*	c.939							
p.309	G	L	L	A	L	p.313							
Mutant													

Human *ATP2C1* gene mutations were summarized. The mutations are grouped for deletion/insertion (azure), nonsense (orange), missense (yellow), acceptor/donor splice (green). Nucleotides are reported in italic along the table as well as in the figure legend and along the full manuscript. We found some reported mutations were inaccurate or not unified. Therefore, we revised or collated some descriptions according to the reported cDNA reference sequence (GenBank accession No. NM_AF181120).^7^

Appendix 1: A polymorphism was wrongly reported to be a new mutation 2323*C*>*T* generating Y711H.^87^ After careful check with the correct reading frame this was not a mutation but a polymorphism.^88^

Appendix 2: A resuming panel graphs the amount and relative percentage of each kind of mutations and PTC.

Appendix 3: The 884–904del*CCATTGTGGTCACAGTGACGC* mutation and consequent amino acid 296-302delIVVTVTL was incorrectly reported,^18^ while it referred to as a 21bp deletion located at 932-952 and amino acid 311-317delPIVVTVT. This mutation did not generate the reported missense mutation P295V;^18^ the first ‘*C*' of the codon 311 (encoding for a proline, P) recombined with ‘*TA*' of codon 318 (leucine, L) generating the codon *CTA* which encoded for a leucine.

a Number of reported cases of patients presenting the mutation.

b Missense mutations causing an amino acid substitution in extremely conserved residue through all the ATPases and in different species are highlighted in light pink.^23^

c Putative protein domain prediction is based on the position of the equivalent residue within the structure of ATP2A1 (SERCA1).

d Using the running correct coding sequence and relative reading frame of the *ATP2C1* gene (Ref. NG_007379.1) we unified the position of the mutation site, protein change, exon/intron location all over the reported mutations. In doing this we found few mutations published as new which were already known.

e The same authors published their findings in two identical papers on different journals.^22, 89^

f Due to incorrect interpretation of discovered mutations the authors reported as new previously reported mutations.^90^

g In their manuscript, the authors reported a previously described mutation. A mistake on referring to this mutation was recently reported (see erratum in *Acta Derm Venereol* 2015; 95: 1040).

h P201L, C344Y, and T570I, respectively, represent mutations P185L, C328Y, and T554I, originally reported by Sudbrak *et al.*^6^ Mutation nomenclature has now been updated with respect to the 5'-end sequence published by Hu *et al*^7^ and the results of 5' RACE-PCR experiments from Fairclough *et al.*^36^

i The missense mutation 832*G*>*A* causing the nucleotide change G278R generated an aberrant splicing with a resulting insertion of the first 11 bp (*GTAAGAGAAGA*) from intron 10 between the mutated exon10 and the exon 11 (see Figure 5 in Chao *et al*)^91^ causing a PTC.

j This mutation was incorrectly reported as A304T by Hu *et al*^7^ as previously reported.^20^

k E308, and not G309 (which are both Ca2+-binding site residue), was deleted.

l 950del*CGCTAGCTCTT*>*CT*>ins*CCACAATGTGTTGGTGTTATGAGAAT* (underlined are the deleted/inserted nucleotides) generates the in frame 318delLAL/insTMCWCYEN.

m 1068delAAGAATGAAATGACTG.

n The del*GACAGACCAGAGATTTGTTTTATGAAAG* cause a frame shift and PTC.

o In frame del*AAGTACTGTACTACATACCAGAGC* with amino acid delKYCTTYQS.

p This mutation was incorrectly reported as P724R by Hu *et al*^7^ as previously reported.^20^

q The deleted sequence is *TGGGACAATT*.

## Abbreviations

CASQ: calsequestrin
COPI: coat protein complex I
HHD: Hailey–Hailey disease
IGF1R: insulin-like growth factor receptor
ER: endoplasmic reticulum
PMR1: the yeast homolog of SPCA1
SERCA: ER ATPase Ca^2+^ pump type 2
SPCA1: secretory pathway Ca^2+^-ATPase pump type 1
TGN: *trans*-Golgi network
