# Supplementary material for: ATP2C1 gene mutations in Hailey–Hailey disease and possible roles of SPCA1 isoforms in membrane trafficking
Source: Cell Death Dis. 2016 Jun 9;7(6):e2259–. doi: 10.1038/cddis.2016.147 (PMC5143377; doi:10.1038/cddis.2016.147)
Supplement: Supplementary Figure 1 [file cddis2016147x1.pdf]

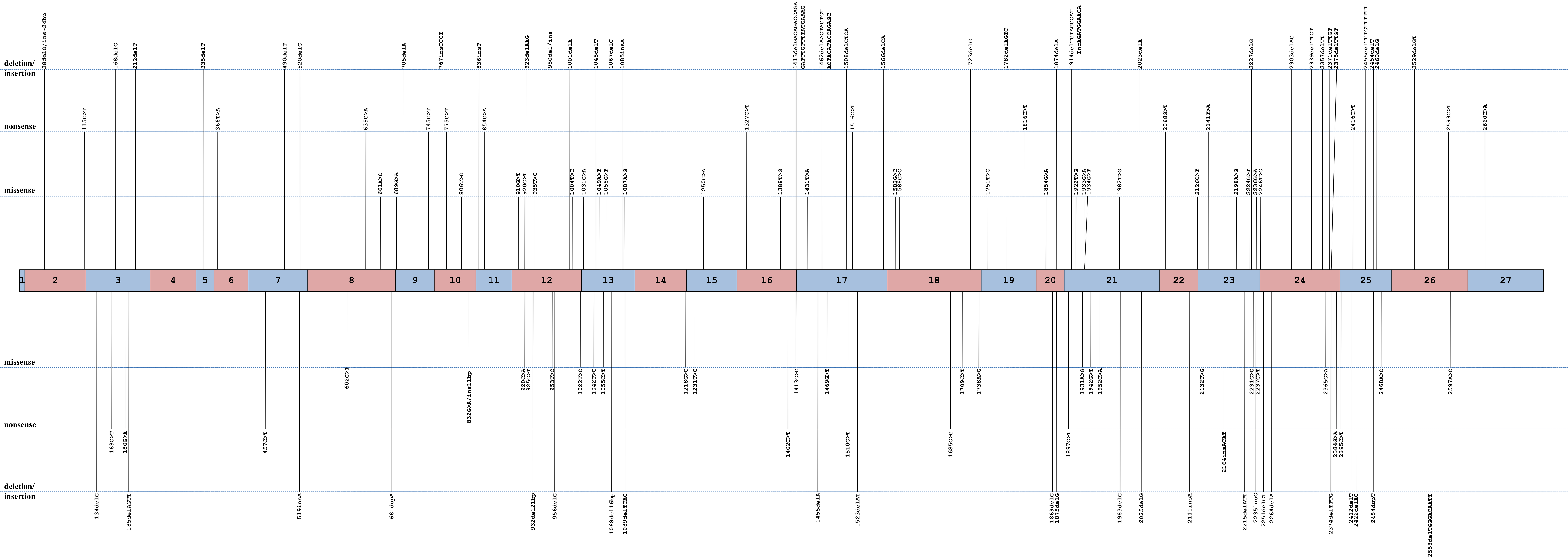

deletion/ insertion
nonsense
missense
missense
nonsense
deletion/ insertion
28delG/ins~24bp
168delC
212delT
335delT
490delT
520delC
705delA
767insCCCT
836insT
923delAAG
950del/ins
1001delA
1045delT
1067delC
1085insA
1413delGACAGACCAGA GATTTGTTTATGAAAG
1462delAAGTACTGT ACTACATACCAGAGC
1508delCTCA
1566delCA
1723delG
1782delAGTC
1874delA
1914delTGTAGCCAT InsAGATGGAACA
2023delA
2227delG
2303delAC
2339delTTGT
2357delTT
2371delTTGT
2375delTTGT
2455delTGTGTTTTTT
2454delT
2460delG
2529delGT
115C>T
366T>A
635C>A
745C>T
775C>T
854G>A
1327C>T
1516C>T
1816C>T
2068G>T
2141T>A
2416C>T
2593C>T
2660C>A
661A>C
689G>A
806T>G
910G>T
920C>T
935T>C
1004T>C
1031G>A
1049A>T
1058G>T
1087A>G
1250G>A
1388T>G
1431T>A
1582G>C
1588G>C
1751T>C
1854G>A
1922T>G
1933G>A
1934G>T
1982T>G
2126C>T
2198A>G
2224G>T
2236G>A
2246T>G
1 2 3 4 5 6 7 8 9 10 11 12 13 14 15 16 17 18 19 20 21 22 23 24 25 26 27
602C>T
832G>A/ins11bp
920C>A
925G>T
953T>C
1022T>C
1042T>C
1055C>T
1218G>C
1231T>C
1413G>C
1469G>T
1709C>T
1738A>G
1931A>G
1942G>T
1952C>A
2132T>G
2231C>G
2237C>T
2365G>A
2468A>C
2597A>C
163C>T
180G>A
457C>T
1402C>T
1510C>T
1685C>G
1897C>T
2164insACAT
2384G>A
2395C>T
134delG
185delAGTT
519insA
681dupA
932del21bp
956delC
1068del16bp
1089delTCAC
1455delA
1523delAT
1869delG
1875delG
1983delG
2025delG
2111insA
2215delATT
2235insC
2251delGT
2264delA
2374delTTTG
2412delT
2422delAC
2454dupT
2558delTGGGACAATT
